# Synergistic Promotion of Somatic Embryogenesis in *Pinus elliottii* by Reduced Glutathione and Riboflavin

**DOI:** 10.3390/plants15182768

**Published:** 2026-09-10

**Authors:** Lin Xu, Wei Ding, Xi Ouyang, Bin Gong, Qian Liu, Zhaolei Deng, Chunxia Yang

**Affiliations:** 1 Jiangxi Academy of Forestry, Nanchang 330013, China; xulin@njfu.edu.cn (L.X.); njfu_ding@126.com (W.D.); oyxaccounting@sina.com (X.O.); gongbin0505@163.com (B.G.); lq_lky18670223641@126.com (Q.L.); dengzhaolei97@126.com (Z.D.); 2 Jiangxi Provincial Key Laboratory of Improved Variety Breeding and Efficient Utilization of Native Tree Species, Nanchang 330013, China; 3 Key Laboratory of National Forestry and Grassland Administration on Forest Cultivation and Management in East China, Nanchang 330013, China

**Keywords:** *Pinus elliottii*, somatic embryogenesis, reduced glutathione, riboflavin, synergistic effect, normal embryo ratio

## Abstract

Somatic embryogenesis (SE) is a core technology for the clonal propagation of forest tree species. However, the low frequency of normal embryos and high rate of abnormal embryos during SE maturation induction in slash pine (*Pinus elliottii*) severely constrain its commercial application. In this study, comparative transcriptome analysis between normal embryos (NE) and abnormal embryos (AE) revealed that glutathione metabolism and riboflavin metabolism pathways may be involved in the regulation of somatic embryo maturation. Based on these findings, different concentration gradients of reduced glutathione (GSH) and riboflavin were supplemented into the somatic embryo maturation medium to verify their effects. Single-factor experiments showed that the 0.5 mM GSH treatment yielded 158.33 normal embryos per gram of embryogenic callus with a normal embryo ratio of 42.20%, while the 7.5 µM riboflavin treatment yielded 100 normal embryos per gram with a normal embryo ratio of 18.27%. Further 2 × 2 orthogonal combination experiments demonstrated that the synergistic treatment of 0.5 mM GSH and 7.5 µM riboflavin achieved the optimal effect, producing 333.33 normal embryos per gram of embryogenic callus with a normal embryo ratio of 72.88%. Both maturation rate and induction efficiency were significantly superior to single treatments and other combinations. These results demonstrate that GSH and riboflavin can synergistically improve the normal embryo ratio and accelerate somatic embryo maturation in slash pine. The underlying mechanisms involving metabolic and hormonal pathways warrant further physiological and biochemical investigation. This study provides a technical reference for optimizing the somatic embryogenesis system of slash pine.

## 1. Introduction

Slash pine (*Pinus elliottii*) is an important industrial timber and ecological protection forest species in southern China, with substantial market demand for superior varieties. However, traditional breeding programs require 15–30 years per cycle, making it difficult to meet the urgent needs of industrialized forestry development [1]. Somatic embryogenesis (SE), as a core technology for clonal propagation of improved forest tree varieties, enables the fixation of total genetic gains from elite genotypes within a single generation, representing a critical pathway for forest tree improvement [2]. Nevertheless, despite significant advances in SE technology for coniferous species, the large-scale application in slash pine remains constrained by efficiency bottlenecks during the maturation phase [3,4]. The SE process comprises three sequential stages: induction/proliferation, maturation, and germination, among which the maturation stage constitutes the primary limiting factor for overall efficiency. This stage is characterized by low normal embryo frequency, high abnormal embryo rates, and strong genotype dependence [5]. Notably, the maturation efficiency of SE in coniferous species is generally inferior to that in broadleaf trees and agricultural crops; therefore, optimizing the somatic embryo maturation protocol and improving normal embryo frequency in slash pine hold significant theoretical value and application prospects [6].

Currently, conifer somatic embryo maturation cultures rely primarily on exogenous abscisic acid (ABA) as the core inductive agent, supplemented with osmotic regulators such as polyethylene glycol (PEG) or elevated sucrose concentrations [7,8]. Although ABA is indispensable for promoting embryo desiccation tolerance and storage product accumulation, its efficacy in improving normal embryo frequency remains limited [9,10]. Osmotic regulators can improve embryo quality to some extent, but supraoptimal PEG concentrations readily induce vitrification or developmental arrest [11]. Activated charcoal, commonly employed to mitigate oxidative browning and adsorb inhibitory metabolites, suffers from insufficient concentration optimization—excessive amounts may adsorb essential nutrients, whereas insufficient supplementation fails to effectively prevent browning [12]. The interplay of these factors results in poor developmental synchrony and inconsistent embryo quality under existing culture conditions, necessitating novel approaches to explore maturation regulation strategies.

Existing SE maturation research has focused predominantly on hormonal and osmotic regulation, with inadequate attention to the systematic modulation of intracellular metabolic states [13]. Glutathione (GSH) is the most abundant non-enzymatic antioxidant in plant cells, directly participating in reactive oxygen species scavenging and redox homeostasis maintenance. The GSH redox state (GSH/oxidized glutathione (GSSG) ratio) has been demonstrated to influence cell division and differentiation in various plants [14,15]. However, direct evidence for the role of GSH in conifer somatic embryo maturation remains lacking. Riboflavin (vitamin B_2_) serves as the precursor of flavin mononucleotide (FMN) and flavin adenine dinucleotide (FAD), which function as core cofactors in the mitochondrial electron transport chain and determine cellular energy supply efficiency [16]; riboflavin metabolic disorders lead to embryonic developmental arrest, yet the regulatory function of riboflavin metabolism in SE has scarcely been investigated in forest trees [17]. More critically, GSH-mediated redox homeostasis and riboflavin-driven energy metabolism do not operate independently—the redox state affects the activity of FMN/FAD-dependent enzymes, while energy supply determines GSH regeneration rate; however, the synergistic effect of these two pathways in somatic embryo maturation has not been reported to date [18,19].

In this study, we used slash pine as the experimental material to: (i) compare the transcriptomic differences between normal embryos (NE) and abnormal embryos (AE) to identify metabolic pathways associated with embryo developmental quality; (ii) verify the independent regulatory effects and optimal concentrations of exogenous GSH and riboflavin on somatic embryo maturation; and (iii) explore the feasibility of synergistic interactions between the two compounds, thereby providing technical parameters and theoretical foundations for establishing an efficient somatic embryo maturation protocol for slash pine.

## 2. Results

### 2.1. Differential Gene Expression Between AE and NE

**Figure 1:**
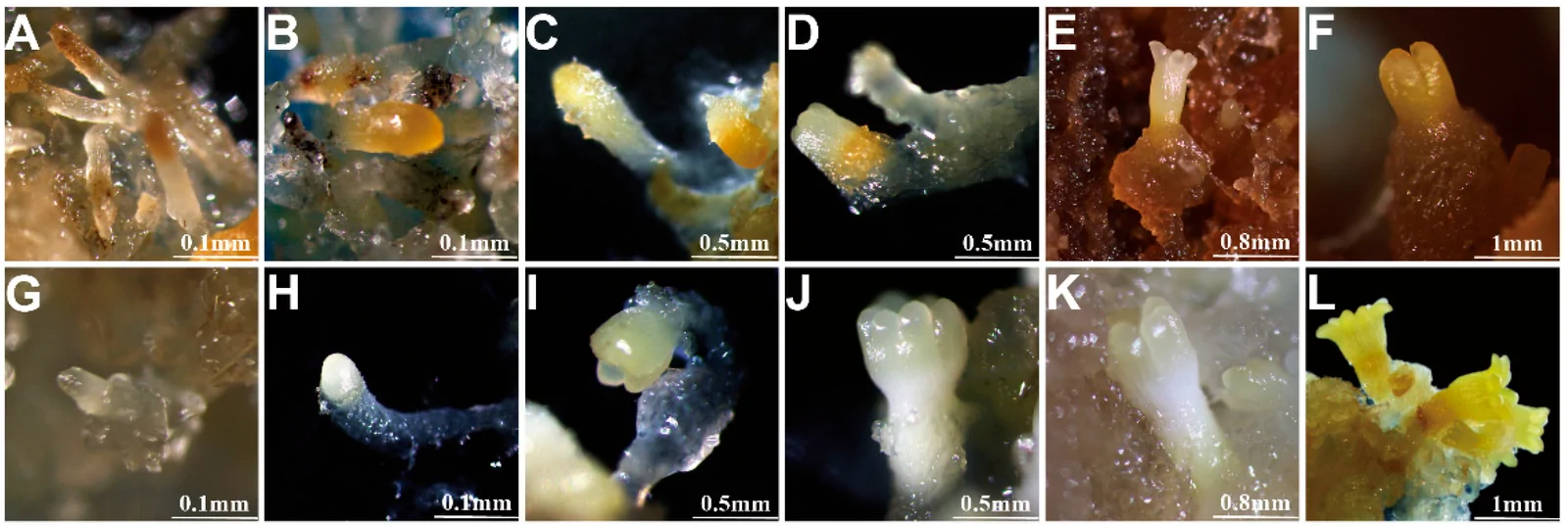
Phenotypic observation of AE and NE in slash pine. (**A**–**F**): Morphological observation of AE. (**A**,**B**), early developmental stage. (**C**,**D**), mid developmental stage. (**E**,**F**), late developmental stage. (**G**–**L**): Morphological observation of NE. (**G**,**H**), early developmental stage. (**I**,**J**), mid developmental stage. (**K**,**L**), late developmental stage.

In this study, to elucidate the molecular mechanisms underlying embryo development during somatic embryogenesis maturation in slash pine and address the technical bottleneck of low normal embryo frequency, we conducted a comparative transcriptomic analysis between NE and AE. Based on morphological characteristics, embryos were classified as follows: NE exhibited normal coloration (translucent at early stage, milky white at mid-stage, and light green at late stage), absence of browning or vitrification, ≥3 cotyledons, smooth hypocotyls with distinct boundaries, and potential for continued development (Figure 1G–L); embryos failing to meet these morphological criteria were categorized as AE, which typically arrested development due to browning or structural defects (Figure 1A–F).

**Figure 2:**
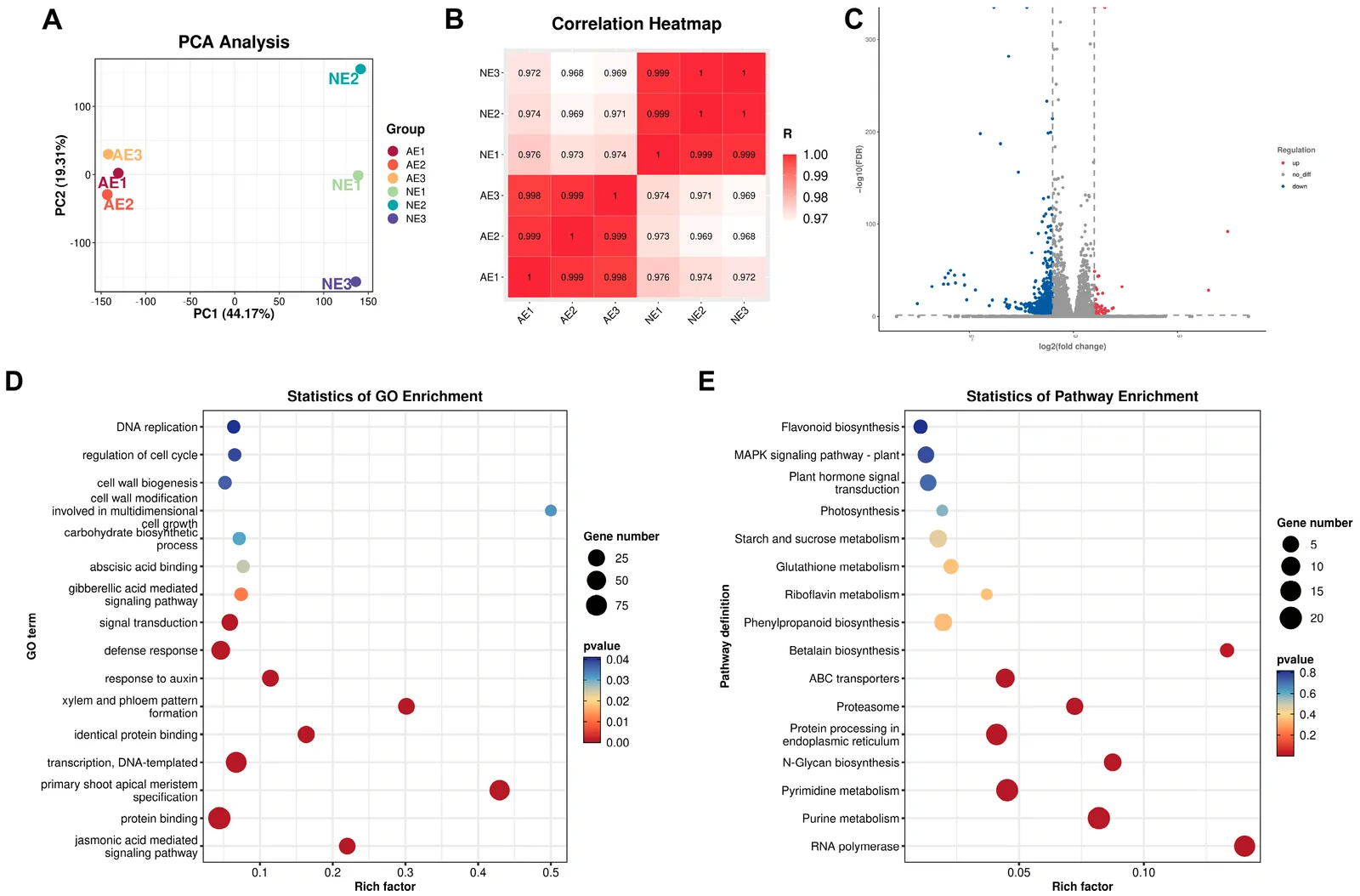
Transcriptomic differential analysis between AE and NE. (**A**): Principal component analysis (PCA). (**B**): Inter-sample correlation heatmap. (**C**): Volcano plot of differentially expressed genes. (**D**): GO functional enrichment analysis. (**E**): KEGG pathway enrichment analysis.

To reveal the transcriptional expression differences between AE and NE, we performed principal component analysis (PCA) and inter-sample correlation analysis on variance-stabilized count data (Figure 2A,B). PCA demonstrated clear separation among samples, with all samples clustering into two distinct groups (Figure 2A). The inter-sample correlation heatmap indicated high consistency within biological replicates (Figure 2B). Differentially expressed genes (DEGs) were identified using the following criteria: |log_2_(fold change)| > 1, Q-value < 0.05, and TPM > 1. Compared with AE, a total of 64 genes were upregulated and 1225 genes were downregulated in NE (Figure 2C).

Functional enrichment analysis of DEGs was performed. GO enrichment results showed that differential genes were mainly enriched in plant hormone-related terms, including jasmonic acid mediated signaling pathway (GO:0009867), response to auxin (GO:0009733), gibberellic acid mediated signaling pathway (GO:0009740), and abscisic acid binding (GO:0010427), as well as plant growth and development-related terms such as primary shoot apical meristem specification (GO:0010072), defense response (GO:0006952), signal transduction (GO:0007165), and regulation of cell cycle (GO:0051726) (Figure 2D). These results suggest that alterations in plant hormone signaling networks and genes regulating cell division and differentiation may be important contributors to abnormal somatic embryo development in slash pine. KEGG enrichment analysis revealed that differential genes were significantly enriched in RNA polymerase (map03020), purine metabolism (map00230), pyrimidine metabolism (map00240), and betalain biosynthesis (map00965) pathways. Additionally, DEGs were also enriched in riboflavin metabolism (map00740) and glutathione metabolism (map00480), indicating that disturbances in energy metabolism, nucleic acid synthesis, and redox homeostasis may participate in the regulation of normal somatic embryo development and maturation in slash pine (Figure 2E).

### 2.2. Differential Gene Expression Analysis

#### 2.2.1. Plant Hormone Signaling-Related Gene Analysis

**Figure 3:**
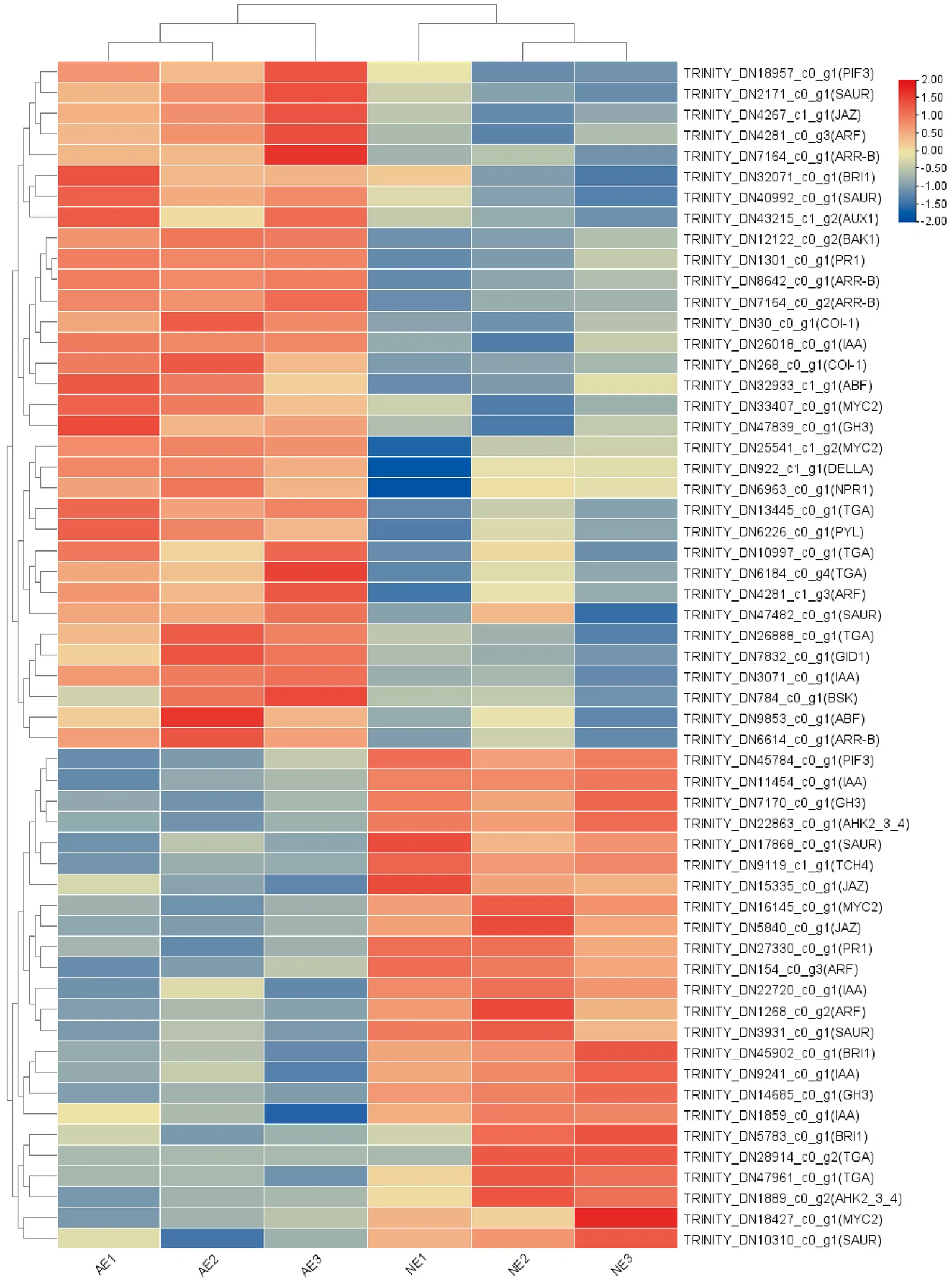
Heatmaps of plant hormone signal transduction pathway genes.

Differentially expressed genes involved in plant hormone signal transduction pathways were extracted based on KEGG and GO annotations and visualized as heatmaps (Figure 3). The results revealed that certain differentially expressed genes participate in the canonical “TIR1/AFB–AUX/IAA–ARFs” auxin signaling pathway. Members of four gene families—*ARF*, *AUX/IAA*, *GH3*, and *SAUR*—showed differential expression between NE and AE, with distinct homologs within the same family exhibiting opposing expression trends. Specifically, some homologs were upregulated in NE (e.g., *ARF*: TRINITY_DN154_c0_g3, TRINITY_DN1268_c0_g2; *AUX/IAA*: TRINITY_DN1859_c0_g1), whereas others were upregulated in AE (e.g., *ARF*: TRINITY_DN4281_c0_g3, TRINITY_DN4281_c1_g3; *AUX/IAA*: TRINITY_DN3071_c0_g1). This divergent expression pattern may reflect functional complementation among homologous genes. These findings indicate significant differences in auxin signal transduction between NE and AE, suggesting that the regulatory state of the auxin pathway is closely associated with the normal developmental competence of somatic embryos.

**Figure 4:**
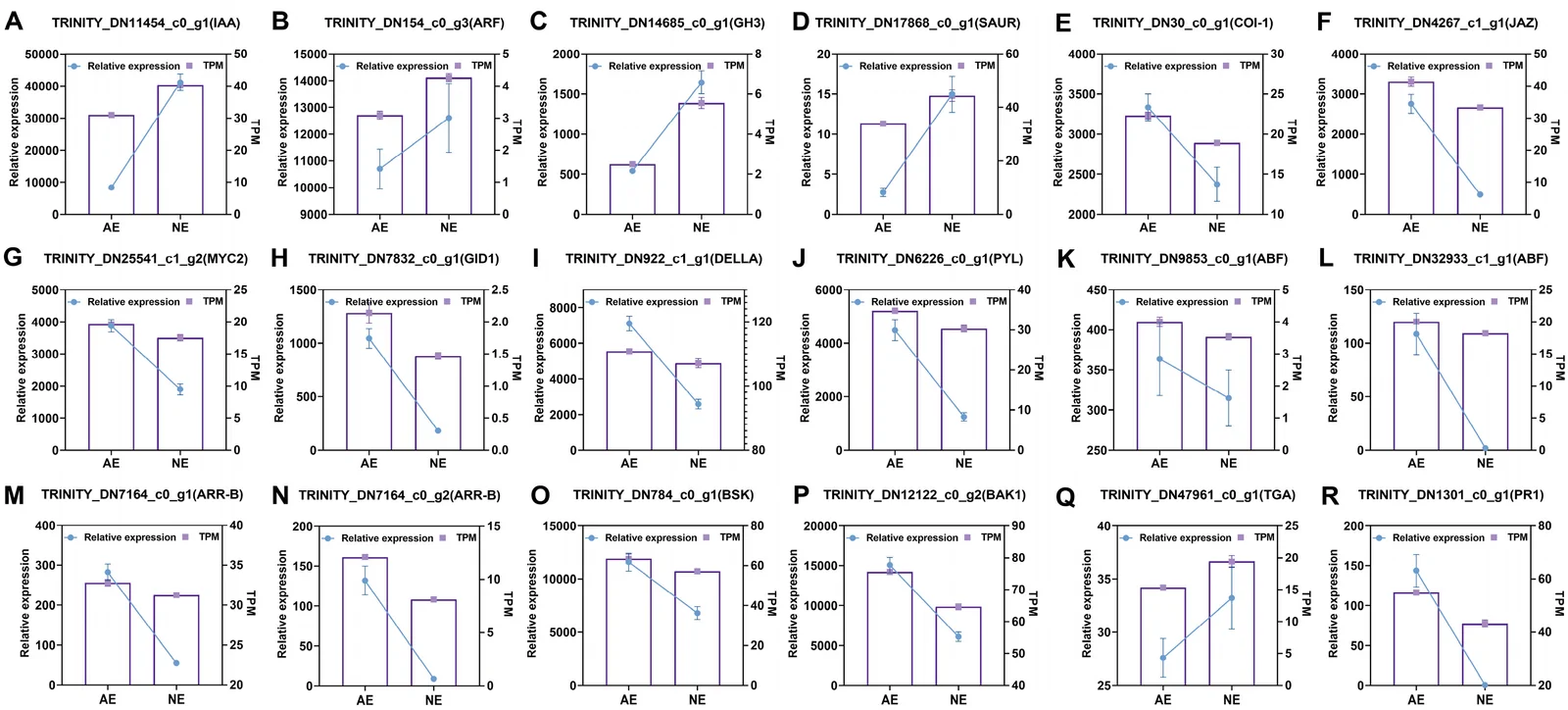
Validation of gene expression in plant hormone signal transduction pathways. (**A**–**D**): Expression verification of key genes in the auxin signal transduction pathway. (**E**–**G**): Expression verification of key genes in the JA signal transduction pathway. (**H**,**I**): Expression verification of key genes in the GA signal transduction pathway. (**J**–**L**): Expression verification of key genes in the ABA signal transduction pathway. (**M**,**N**): Expression verification of key genes in the CK signal transduction pathway. (**O**,**P**): Expression verification of key genes in the BR signal transduction pathway. (**Q**,**R**): Expression verification of key genes in the SA signal transduction pathway. Blue lines indicate qRT-PCR results, purple bars indicate transcriptome sequencing results, and error bars represent standard error (SEM).

Furthermore, homologs involved in the jasmonate (JA) signaling pathway [*JAZ* (TRINITY_DN15335_c0_g1, TRINITY_DN5840_c0_g1) and *MYC2* (TRINITY_DN16145_c0_g1)], gibberellin (GA) signaling pathway [*PIF3* (TRINITY_DN45784_c0_g1)], cytokinin (CK) signaling pathway [*CRE1* (TRINITY_DN1889_c0_g2, TRINITY_DN22863_c0_g1)], brassinosteroid (BR) signaling pathway [*BRI1* (TRINITY_DN45902_c0_g1) and *TCH4* (TRINITY_DN9119_c1_g1)], and salicylic acid (SA) signaling pathway [*TGA* (TRINITY_DN47961_c0_g1) and *PR1* (TRINITY_DN27330_c0_g1)] were upregulated in NE. In contrast, homologs involved in the ABA signaling pathway [*PYL* (TRINITY_DN6226_c0_g1) and *ABF* (TRINITY_DN9853_c0_g1, TRINITY_DN32933_c1_g1)] were upregulated in AE (Figure 3). These results suggest that the expression levels of *JAZ* and 19 other homologs are positively correlated with normal embryo development capacity, whereas *PYL* and 31 other genes show a negative correlation. Selected genes from each hormone signaling pathway were further validated by qRT-PCR, and their expression trends were consistent with the transcriptome sequencing data (Figure 4). In summary, multiple plant hormone signal transduction-related genes exhibited differential expression between NE and AE, indicating that these genes may coordinately participate in the regulation of somatic embryo maturation in slash pine, laying the foundation for subsequent developmental processes.

#### 2.2.2. Expression Analysis of Key Somatic Embryogenesis Genes

**Figure 5:**
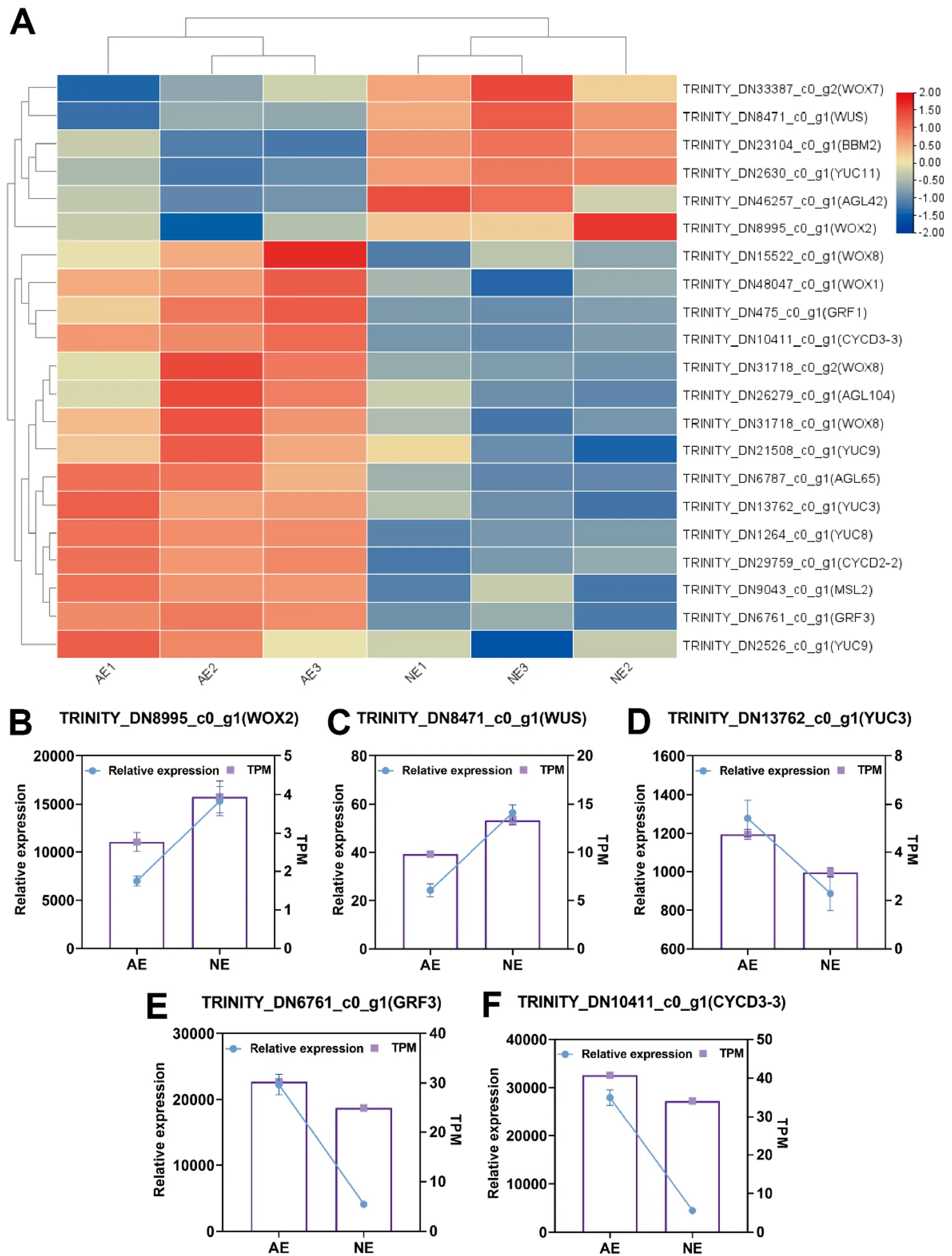
Expression of key genes involved in somatic embryogenesis. (**A**): Heatmap of key somatic embryogenesis genes. (**B**–**F**): Expression verification of key somatic embryogenesis genes. Blue lines indicate qRT-PCR results, purple bars indicate transcriptome sequencing results, and error bars represent standard error (SEM).

To further investigate whether differential expression of key somatic embryogenesis genes between AE and NE is associated with normal embryo development, members of seven gene families related to somatic embryogenesis—*WOX*, *AGL*, *BBM*, *YUC*, *GRF*, *MSL*, and *CYCD*—were identified and their expression patterns in AE and NE were analyzed (Figure 5). Transcriptome data showed that *WUS* homolog (TRINITY_DN8471_c0_g1), *WOX2* homolog (TRINITY_DN8995_c0_g1), *WOX7* homolog (TRINITY_DN33387_c0_g2), *BBM2* homolog (TRINITY_DN23104_c0_g1), *AGL42* homolog (TRINITY_DN46257_c0_g1), and *YUC11* homolog (TRINITY_DN2630_c0_g1) were upregulated in NE (Figure 5A). The expression trends of these six genes were positively correlated with normal embryo developmental competence, suggesting their potential as candidate marker genes for evaluating somatic embryogenesis potential in slash pine. Selected key genes were further validated by qRT-PCR, and their expression trends were consistent with the transcriptome sequencing results (Figure 5B–F), confirming the reliability of the observed differential expression patterns.

#### 2.2.3. Expression Analysis of Key Genes in Riboflavin Metabolism

**Figure 6:**
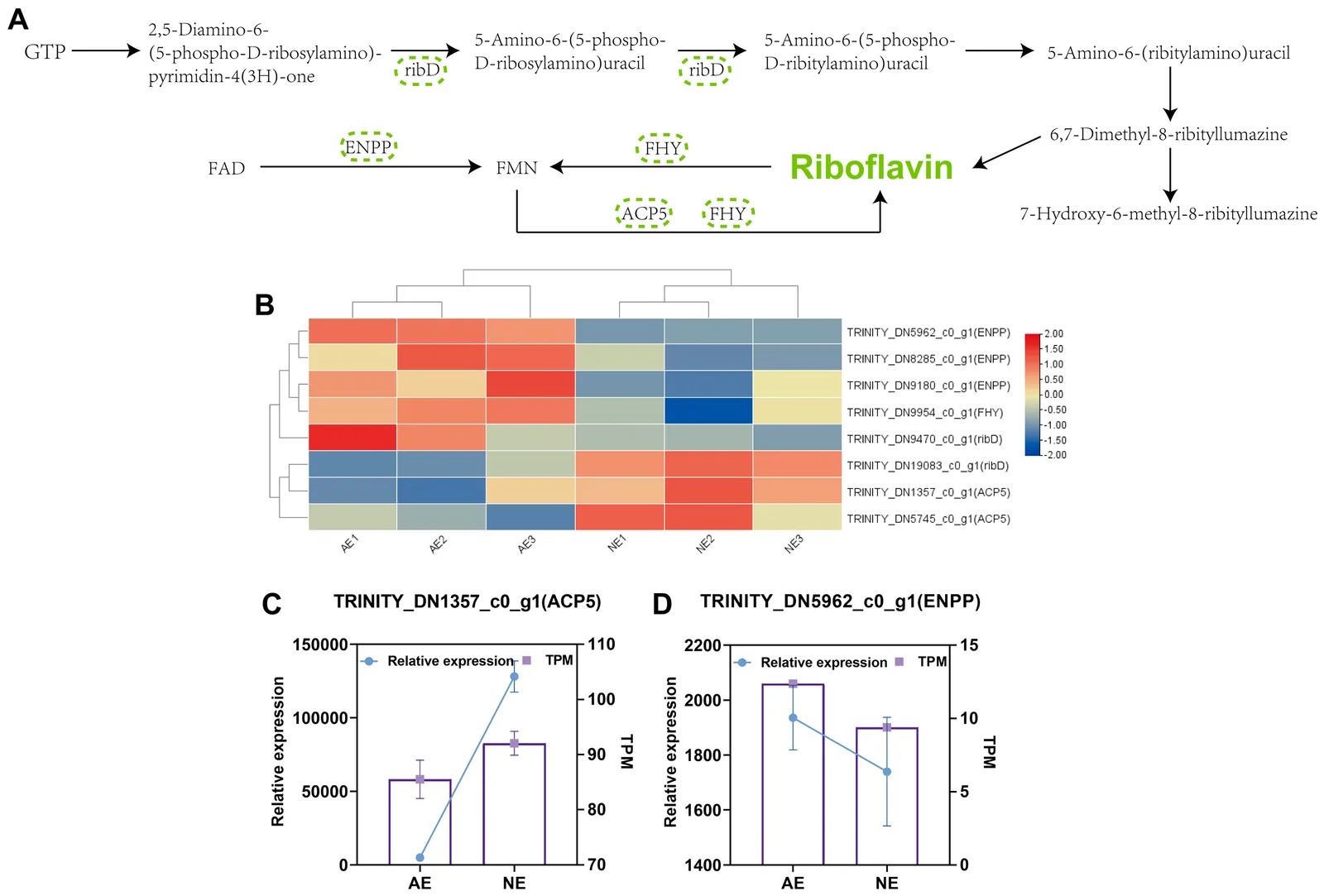
Expression analysis of key genes in riboflavin metabolism. (**A**): Schematic diagram of riboflavin metabolism. (**B**): Heatmap of key genes in riboflavin metabolism. (**C**,**D**): Expression verification of key genes in riboflavin metabolism. Blue lines indicate qRT-PCR results, purple bars indicate transcriptome sequencing results, and error bars represent standard error (SEM).

Riboflavin metabolism may play an important role in somatic embryogenesis. Comparison of gene expression profiles in this pathway between AE and NE revealed that *ribD* homolog (TRINITY_DN19083_c0_g1) and *ACP5* homologs (TRINITY_DN1357_c0_g1, TRINITY_DN5745_c0_g1) were upregulated in NE (Figure 6B). *ribD* encodes the rate-limiting enzyme in riboflavin biosynthesis, catalyzing the conversion of GTP to riboflavin precursors; *ACP5* is involved in acyl carrier protein metabolism, which is closely associated with cellular energy supply. The elevated expression of these genes in NE may enhance the synthesis efficiency of riboflavin cofactors (FMN/FAD), optimize mitochondrial electron transport function and cellular energy metabolic status, thereby providing necessary energy support for normal embryo development (Figure 6A). qRT-PCR validation results were consistent with the transcriptome data trends (Figure 6C,D), further confirming the involvement of riboflavin metabolism in the regulatory mechanism of somatic embryo maturation induction in slash pine.

#### 2.2.4. Expression Analysis of Key Genes in Glutathione Metabolism

**Figure 7:**
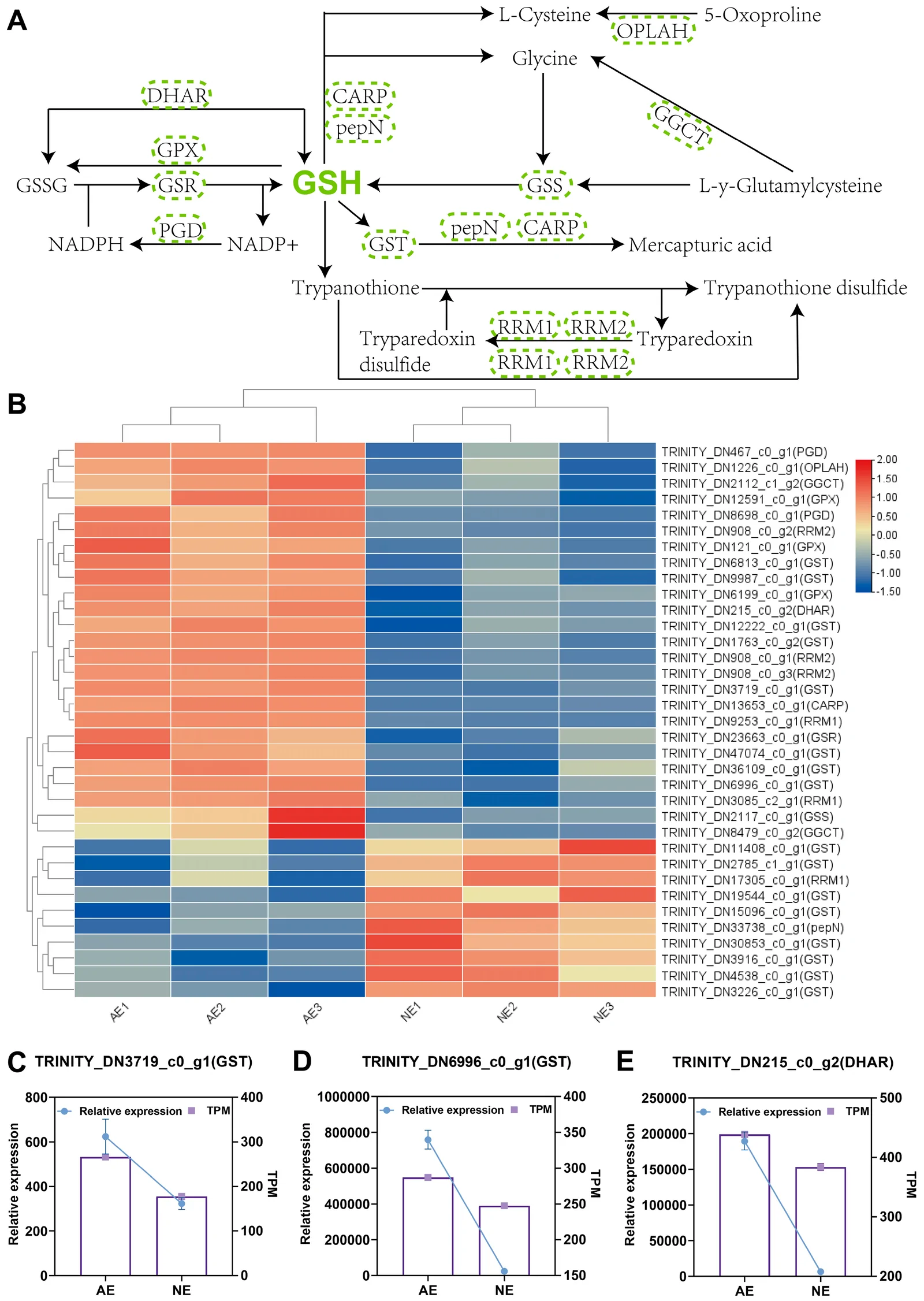
Expression analysis of key genes in glutathione metabolism. (**A**): Schematic diagram of glutathione metabolism. (**B**): Heatmap of key genes in glutathione metabolism. (**C**–**E**): Expression verification of key genes in glutathione metabolism. Blue lines indicate qRT-PCR results, purple bars indicate transcriptome sequencing results, and error bars represent standard error (SEM).

The potential role of glutathione metabolism in the regulation of embryogenesis cannot be ignored. Analysis of gene expression patterns in this pathway between AE and NE revealed that *GST* homologs (TRINITY_DN11408_c0_g1, TRINITY_DN2785_c1_g1, TRINITY_DN19544_c0_g1, TRINITY_DN15096_c0_g1, TRINITY_DN30853_c0_g1, TRINITY_DN3916_c0_g1, TRINITY_DN4538_c0_g1, TRINITY_DN3226_c0_g1), *pepN* homolog (TRINITY_DN33738_c0_g1), and *RRM1* homolog (TRINITY_DN17305_c0_g1) were upregulated in NE (Figure 7B), suggesting that these genes may promote normal embryo development by maintaining redox homeostasis (Figure 7A). qRT-PCR validation results were consistent with the transcriptome sequencing trends (Figure 7C–E), further supporting the involvement of the glutathione metabolism pathway in the regulation of somatic embryo maturation induction in slash pine.

### 2.3. Riboflavin Promotes Somatic Embryogenesis in Slash Pine

**Figure 8:**
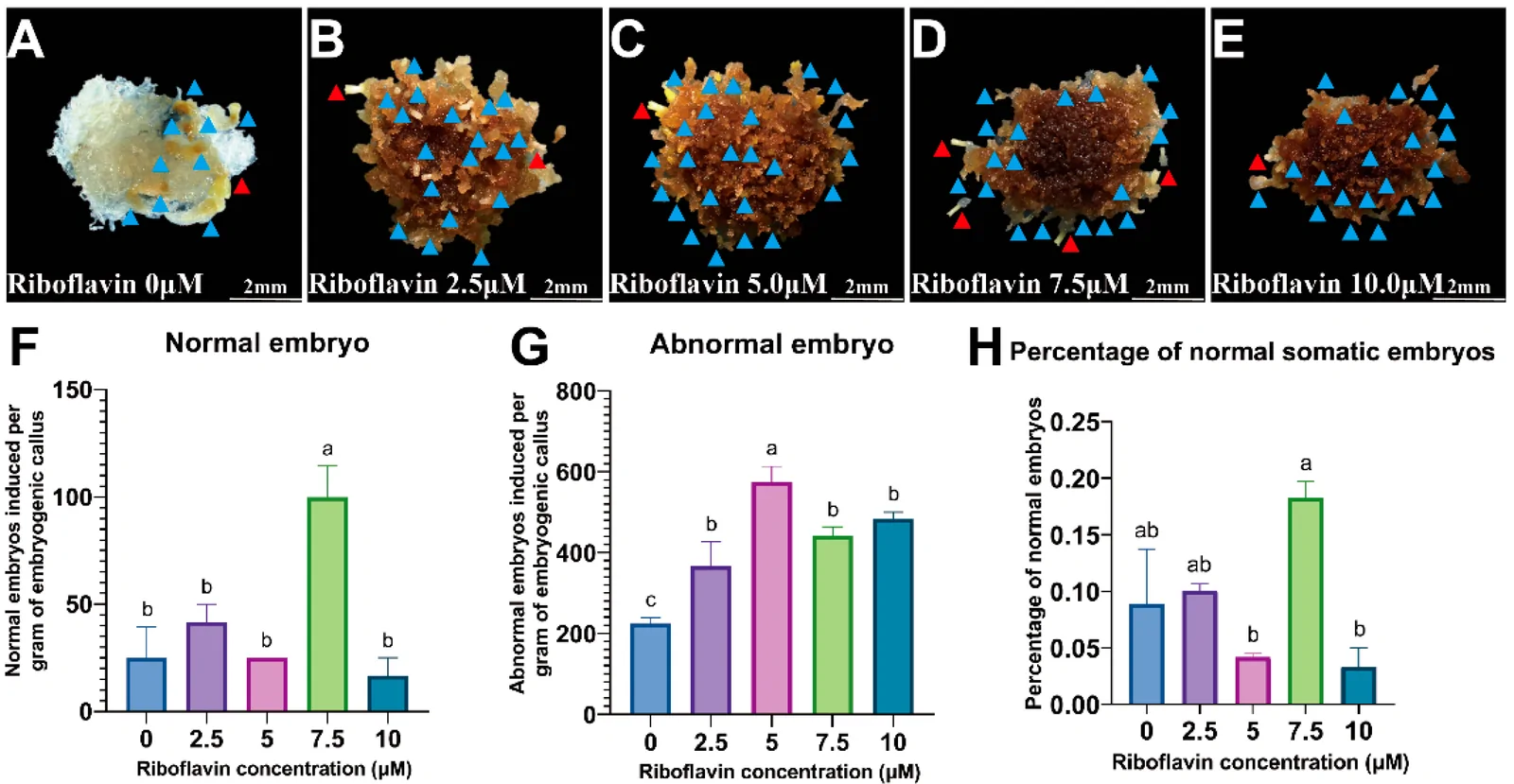
Riboflavin promotes somatic embryogenesis in slash pine. (**A**–**E**): Morphological observation of slash pine embryogenic callus cultured for 120 days in somatic embryo maturation medium with different riboflavin concentrations. (**F**–**H**): Somatic embryo quantity statistics, where (**F**) represents the number of normal embryos, (**G**) represents the number of abnormal embryos, and (**H**) represents the proportion of normal embryos. Different lowercase letters in the same panel indicate significant differences between treatments (*p* < 0.05), while the same letters indicate no significant difference (*p* > 0.05). Red triangles mark NE, and blue triangles mark AE.

Based on the regulatory mechanism of riboflavin metabolism revealed by transcriptome sequencing, we established a series of riboflavin concentration gradients in the somatic embryo maturation induction medium to verify its effects on somatic embryogenesis. After 120 days of induction culture, observation results showed (Figure 8A–E): embryogenic callus cultured in medium containing riboflavin could induce a large number of early embryos, but most embryos in the treatment groups exhibited browning or developmental arrest. Statistical results indicated that riboflavin within the concentration range of ≤7.5 µM promoted somatic embryo maturation and normal embryo development in slash pine, with 7.5 µM identified as the optimal induction concentration—this treatment group produced 100 normal embryos per gram of embryogenic callus, with a normal embryo ratio of up to 18.27%, and the somatic embryo development rate was significantly better than other concentration treatments (*p* < 0.05) (Figure 8F–H). These results demonstrate that exogenous riboflavin at appropriate concentrations can promote somatic embryogenesis in slash pine, but excessive concentrations may lead to abnormal embryo development, necessitating further optimization in subsequent studies.

### 2.4. Reduced Glutathione Promotes Somatic Embryogenesis in Slash Pine

**Figure 9:**
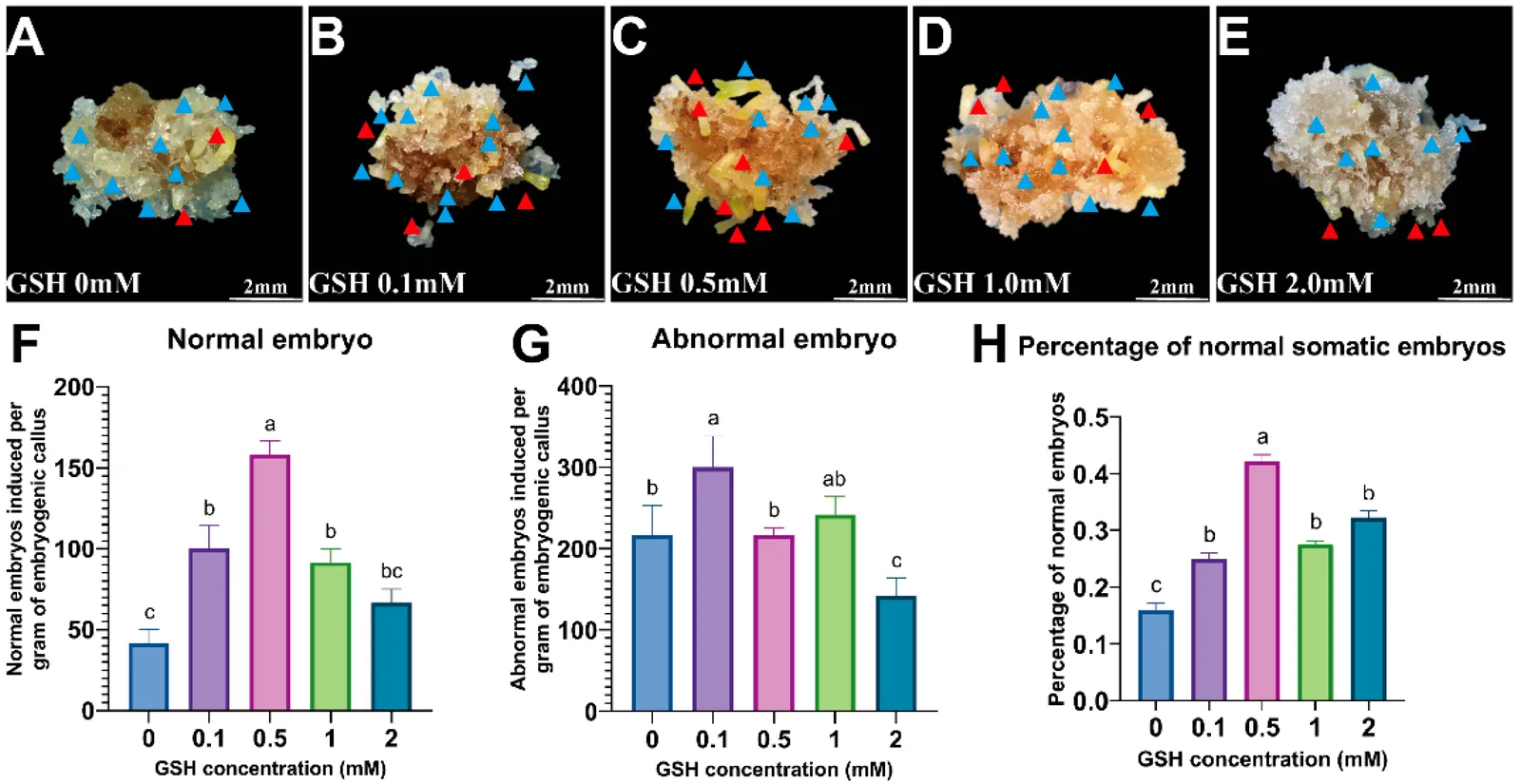
Reduced glutathione promotes somatic embryogenesis in slash pine. (**A**–**E**): Morphological observation of slash pine embryogenic callus cultured for 120 days in somatic embryo maturation medium with different GSH concentrations. (**F**–**H**): Somatic embryo quantity statistics, where (**F**) represents the number of normal embryos, (**G**) represents the number of abnormal embryos, and (**H**) represents the proportion of normal embryos. Different lowercase letters in the same panel indicate significant differences between treatments (*p* < 0.05), while the same letters indicate no significant difference (*p* > 0.05). Red triangles mark NE, and blue triangles mark AE.

Based on the regulatory clues of glutathione metabolism suggested by transcriptome sequencing, we established different concentration gradients of GSH in the somatic embryo maturation induction medium of slash pine to investigate its actual effects on somatic embryogenesis. After 120 days of culture, observation results showed (Figure 9A–E): embryogenic callus cultured in medium containing GSH could induce the production of some mature embryos, and the promoting effect of GSH on development showed obvious concentration dependence. When the GSH concentration was 0.5 mM, it could effectively promote the development of normal somatic embryos in slash pine. This group reached 158.33 normal embryos per gram of embryogenic callus, with a normal embryo ratio as high as 42.20%, and some embryos had completed mature development, with a development rate significantly higher than other concentration treatments (*p* < 0.05) (Figure 9F–H). These results indicate that exogenous GSH at appropriate concentrations can not only promote somatic embryogenesis in slash pine, but also accelerate the somatic embryo development process and improve normal embryo efficiency.

### 2.5. Synergistic Effect of Reduced Glutathione and Riboflavin on Improving Normal Embryo Ratio and Accelerating Somatic Embryo Maturation in Slash Pine

**Figure 10:**
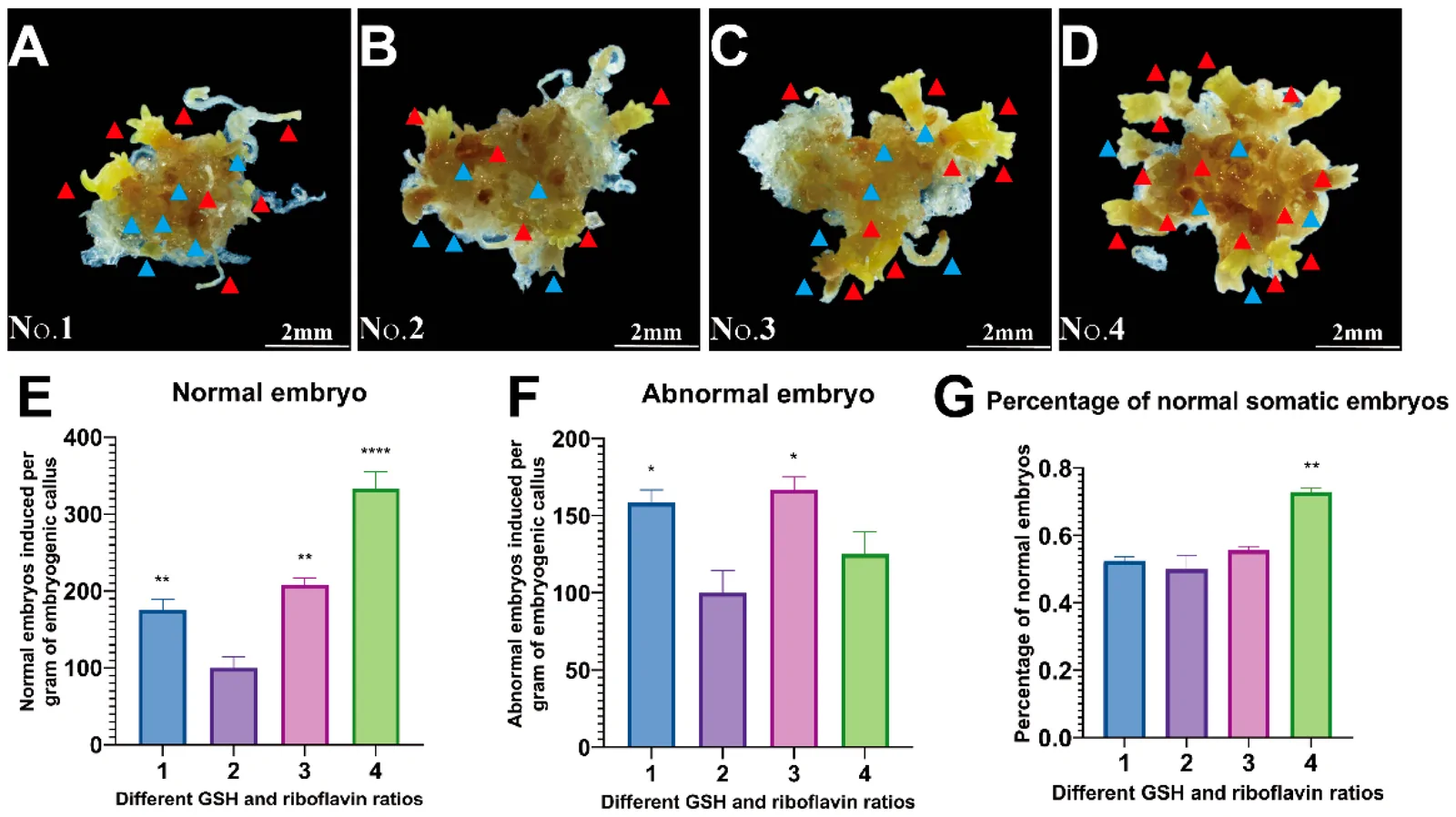
Synergistic promotion of somatic embryogenesis in slash pine by reduced glutathione and riboflavin. (**A**–**D**): Morphological observation of slash pine embryogenic callus cultured for 120 days in somatic embryo maturation medium with different orthogonal combinations. (**E**–**G**): Somatic embryo quantity statistics, where (**E**) represents the number of normal embryos, (**F**) represents the number of abnormal embryos, and (**G**) represents the proportion of normal embryos. Different letters or asterisks in the same panel indicate significant differences between treatments: * *p* < 0.05, ** *p* < 0.01, **** *p* < 0.0001. Red triangles mark NE, and blue triangles mark AE.

Subsequently, we selected two riboflavin concentrations and two GSH concentrations for orthogonal combination, establishing four experimental groups to investigate the synergistic effects of GSH and riboflavin on somatic embryo maturation in slash pine. Morphological observation of embryogenic callus at different time points during somatic embryo induction revealed: at 50 days, a large number of early embryos began to appear on the callus surface; around 80 days, cotyledonary embryos became visible with numerous early embryos continuously emerging; at 100 days, some embryos had matured while others continued to develop; by 120 days, most cotyledonary embryos had matured, producing a large number of morphologically normal mature embryos (Figure 10A–D). Statistical results showed (Figure 10E–G) that the combination of 0.5 mM GSH and 7.5 µM riboflavin achieved the optimal effect, with normal embryo number reaching 333.33 per gram of embryogenic callus (*p* < 0.0001), normal embryo induction ratio as high as 72.88% (*p* < 0.01), and embryogenic callus in good condition with significantly advanced somatic embryo development. Both maturation rate and induction efficiency were significantly superior to other treatment groups. These results demonstrate that GSH and riboflavin at appropriate concentration ratios can synergistically promote the production of normal somatic embryos in slash pine, accelerate the somatic embryo development process, and effectively improve somatic embryo maturation efficiency.

## 3. Discussion

### 3.1. Plant Hormone Signaling Is Associated with Somatic Embryo Developmental Patterns

Differentially expressed genes between NE and AE in this study were enriched in multiple plant hormone signaling pathways, which is consistent with existing research conclusions on conifer somatic embryogenesis. The relatively active ABA signaling pathway in AE warrants in-depth discussion. First, ABA and auxin exhibit antagonistic interactions [20]: in *Arabidopsis thaliana*, ABA can enhance auxin signaling to inhibit hypocotyl elongation [21]. In this study, both auxin- and ABA-related genes were significantly enriched among DEGs, suggesting that shifts in the expression balance of these hormonal pathways are associated with the divergence between NE and AE. However, whether this transcriptomic difference directly causes developmental fate switching, or merely reflects divergent physiological states, cannot be determined from the present data. Second, ABA effects are stage-specific: in *Quercus aliena* Bl., ABA promotes early induction of primary and secondary somatic embryogenesis [22]. The relatively active ABA signaling in slash pine AE may reflect temporal dysregulation. Third, optimal ABA concentrations vary among species: for *Pinus massoniana*, the optimal ABA concentrations for somatic embryo maturation and germination are 2 mg/L and 3 mg/L [23], respectively, whereas slash pine requires 9 mg/L [6], suggesting differences in endogenous synthesis capacity or receptor sensitivity. Collectively, the transcriptional prominence of ABA signaling in AE is associated with morphological phenotypes that include developmental arrest and reduced cotyledon initiation, though a direct causal link remains to be established.

In *Picea abies*, treatment with the polar auxin transport inhibitor N-1-naphthylphthalamic acid (NPA) leads to cotyledon fusion and hypocotyl developmental defects, demonstrating that auxin gradient establishment is central to embryonic pattern formation [24]. The enrichment of auxin response-related GO terms in this study is consistent with a potential role for auxin signaling in slash pine somatic embryo development [25]. Additionally, balance shifts between ABA and GA signaling pathways may also participate in somatic embryogenesis regulation—during wheat seed maturation, ABA-GA balance shifts lead to genetic differences in dormancy induction and maintenance [26]. BR and GA can coordinate rice seed germination and embryonic growth by regulating gluten mobilization [27], indicating that GA functions in embryonic development are conserved across species, although specific mechanisms may vary by species and developmental stage. The co-enrichment of these pathways in our transcriptome dataset suggests that similar hormonal expression patterns may be associated with developmental fate in slash pine, but further functional validation is required to confirm their regulatory roles.

### 3.2. GSH Treatment Is Associated with Improved Somatic Embryo Maturation Efficiency

GSH is an abundant low-molecular-weight thiol that functions in cellular redox buffering and modulates various cellular processes. In other plant systems, GSH-mediated signaling has been linked to reactive oxygen species (ROS) homeostasis [28]. In hybrid larch (*Larix* spp.), GSH metabolism has been reported to be associated with somatic embryogenesis [29]. In *Taxodium* hybrid ‘zhongshanshan’, exogenous GSH treatment was correlated with improved SE efficiency and upregulation of antioxidant-related genes such as *ThGPX4* [30]. In Korean pine (*Pinus koraiensis*), exogenous GSH was reported to promote embryogenic cell proliferation and enhance antioxidant enzyme activity [31]. These studies in other species suggest potential associations between GSH and SE efficiency; however, the present study did not measure ROS levels, antioxidant enzyme activities, or GSH/GSSG ratios. Therefore, while 0.5 mM GSH significantly increased the normal embryo ratio (42.20%) in slash pine, the underlying mechanism—whether through redox modulation, hormone interaction, or other pathways—remains to be experimentally validated.

However, the concentration-dependent effects of GSH vary among different systems. In *Picea pungens*, GSH alone did not improve proliferation efficiency unless combined with 2,4-D, suggesting that GSH effects may depend on concurrent hormonal signals [32]. In the present study, GSH alone promoted maturation-stage embryo development. This difference may reflect stage-specific cellular requirements, though further comparative analysis across proliferation and maturation stages would be needed to confirm this hypothesis.

### 3.3. Riboflavin Treatment Is Associated with Somatic Embryo Maturation

The regulatory function of riboflavin in somatic embryogenesis has only recently begun to receive attention. In longan early somatic embryogenesis studies, miR408 was demonstrated to promote riboflavin biosynthesis by targeting *NUDT23*, thereby elevating m^6^A modification levels and activating cell cycle gene expression. This study also reported that riboflavin metabolism is associated with the pentose phosphate pathway and DNA replication, leading to the proposal that riboflavin may influence cellular metabolic status [33]. However, the present study did not measure ATP levels, mitochondrial function, or metabolic flux, and therefore cannot confirm a direct role for riboflavin in energy metabolism during slash pine somatic embryo maturation.

In this study, exogenous application of 7.5 µM riboflavin yielded 100 normal embryos per gram of embryogenic callus, with a normal embryo ratio of 18.27%. This phenotypic outcome is consistent with the transcriptomic observation that riboflavin metabolism-related genes were differentially expressed between NE and AE, but does not establish a causal mechanism. Whether similar miRNA-mediated regulatory mechanisms exist in slash pine remains unclear, and whether riboflavin affects somatic embryo development through m^6^A modification or other epitranscriptomic mechanisms awaits further investigation.

Additionally, some embryos in the 10.0 µM riboflavin treatment group exhibited browning or developmental arrest, consistent with observations in longan that supraoptimal riboflavin concentrations inhibit cell proliferation. This suggests that riboflavin may exhibit concentration-dependent effects, although optimal concentrations appear to vary among species.

### 3.4. Synergistic Effect of GSH and Riboflavin on Somatic Embryo Maturation

**Figure 11:**
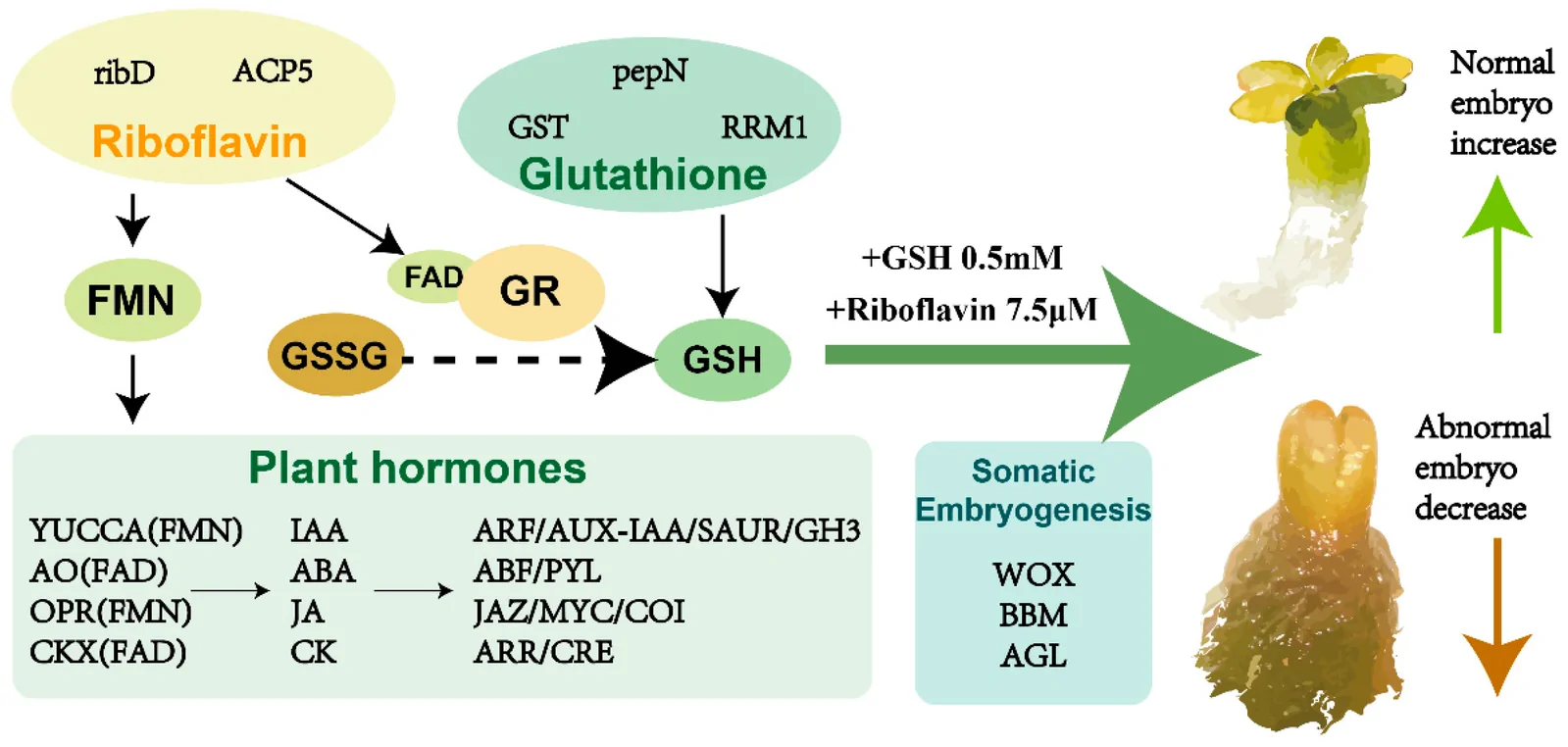
Working hypothesis for the synergistic effect of exogenous GSH and riboflavin on somatic embryo maturation in slash pine. 0.5 mM GSH and 7.5 µM riboflavin regulated GSH metabolism-related genes (GST, pepN, RRM1) and riboflavin metabolism-related genes (ribD, ACP5) in embryogenic cells, respectively (solid arrows, supported by transcriptome data). Based on known flavoprotein biochemistry, we hypothesize that the two pathways may functionally couple at glutathione reductase (GR), where riboflavin-derived FAD serves as the cofactor and GSH metabolism provides the redox substrate (dashed arrows, proposed but not experimentally validated in this study). FMN and FAD are established cofactors for flavoenzymes including YUCCA, AO, OPR, and CKX; we further propose that riboflavin supplementation may influence the catalytic capacity of these enzymes, while GSH may contribute to the redox environment required for flavoenzyme activity. These metabolic changes are hypothesized to be associated with the differential expression patterns of hormone signaling genes observed in normal embryos (ARF/AUX/IAA, JAZ/MYC2, PIF3, CRE1), though direct hormone measurements were not performed. The phenotypic outcome of this synergistic treatment was a normal embryo ratio of 72.88% (333.33 normal embryos per gram embryogenic callus).

The combination treatment of 0.5 mM GSH and 7.5 µM riboflavin in this study produced a significant synergistic effect, with the normal embryo ratio reaching 72.88%, far exceeding single treatments. Based on our transcriptome data and known flavoprotein biochemistry, we propose the following working hypothesis to explain this synergy (Figure 11) [34].

Glutathione reductase (GR) is the key node connecting riboflavin metabolism and GSH metabolism—this enzyme uses FAD as a cofactor to catalyze the reduction in GSSG to regenerate GSH [35]. We hypothesize that riboflavin-derived FAD may support GR activity, while exogenous GSH increases the reduced glutathione pool, potentially creating a cofactor-substrate interaction at the GR level. This proposed coupling is based solely on known enzyme biochemistry and correlative gene expression data; we did not measure GR activity, FAD/FMN contents, or GSH/GSSG ratios in this study. Genetic evidence from *Arabidopsis thaliana* (e.g., embryo-lethal phenotypes of *AtGR2* mutants) is consistent with an important role for GR-mediated GSH regeneration in embryonic development [36], but direct validation in slash pine is lacking. Furthermore, GR and other flavoenzymes such as MDHAR jointly maintain the flux of the AsA-GSH cycle, ensuring ROS scavenging efficiency [37]. The reduced browning observed in the synergistic treatment group is a phenotypic outcome that may be associated with improved cellular redox status; however, we did not quantify ROS levels, antioxidant enzyme activities, or AsA-GSH cycle flux, and therefore cannot attribute the phenotypic improvement to specific antioxidant mechanisms.

Flavoproteins simultaneously participate in the synthesis and metabolism of multiple plant hormones, with their activities dependent on FMN/FAD cofactor supply. The rate-limiting enzyme for auxin synthesis YUCCA, the rate-limiting enzyme for ABA synthesis AO, the key enzyme for JA synthesis OPR, and the cytokinin degradation enzyme CKX are all flavin-dependent enzymes [38,39,40,41]. We hypothesize that riboflavin-derived FMN/FAD may influence the catalytic capacity of these enzymes, while GSH may contribute to the redox environment required for flavoenzyme function. This hypothesis is inferred from gene expression patterns and known enzymatic properties; we did not measure endogenous IAA, ABA, JA, or CK levels, nor did we assay flavoenzyme activities. Thus, the proposed coordination of hormonal balance by GSH and riboflavin remains a mechanistic model awaiting experimental validation.

## 4. Conclusions

In this study, comparative transcriptome analysis between NE and AE of slash pine revealed differential expression of genes associated with glutathione metabolism, riboflavin metabolism, and multiple plant hormone signaling pathways. Based on these transcriptomic clues, we evaluated the independent and combined effects of exogenous GSH and riboflavin on somatic embryo maturation. Single-factor experiments showed that 7.5 µM riboflavin yielded 100 normal embryos per gram of embryogenic callus (18.27% normal embryo ratio), while 0.5 mM GSH yielded 158.33 normal embryos per gram (42.20% normal embryo ratio). A 2 × 2 orthogonal combination revealed that 0.5 mM GSH combined with 7.5 µM riboflavin produced the strongest synergistic effect, achieving 333.33 normal embryos per gram with a normal embryo ratio of 72.88%, significantly outperforming all other treatments. These results demonstrate that the combination of GSH and riboflavin can substantially improve normal embryo frequency and accelerate somatic embryo maturation in slash pine. While our transcriptome data and phenotypic validation support a model in which these metabolites may synergistically influence embryogenic cell status, the precise biochemical mechanisms, including their potential effects on redox balance and energy metabolism, require further physiological and biochemical investigation. This study provides a technical reference for optimizing slash pine somatic embryogenesis and a foundation for future mechanistic studies.

## 5. Materials and Methods

### 5.1. Plant Materials

**Figure 12:**
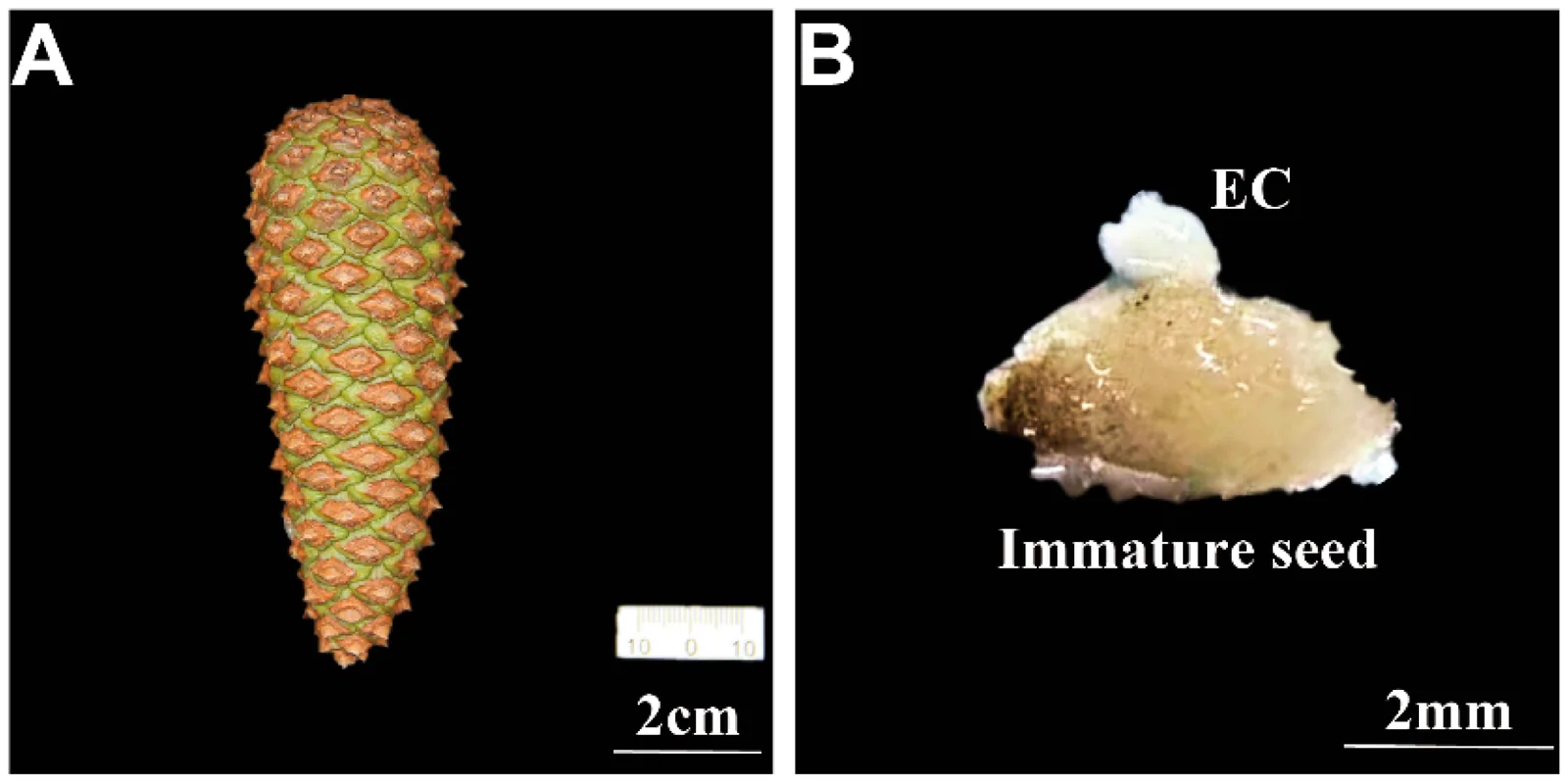
Induction of embryogenic callus in slash pine. (**A**): Immature cones of slash pine. (**B**): Embryogenic callus induced from immature zygotic embryos of slash pine.

The plant material used in this study was immature seeds of slash pine, genotype Sl01-24, collected from the nursery of Jiangxi Academy of Forestry, Nanchang, Jiangxi Province (115°49′3.36″ E, 28°44′20.40″ N). All seeds were collected from open-pollinated mother trees and stored at 4 °C after collection to maintain seed viability (Figure 12A). Seed sterilization was performed in a laminar flow hood: surface disinfection with 75% ethanol for 30 s, followed by three rinses with sterile distilled water; transfer to 10% sodium hypochlorite solution with shaking for 10 min to ensure thorough contact between the disinfectant and seed surface; five rinses with sterile distilled water. Sterilized seeds were blotted dry on sterile filter paper, and the seed coat and endosperm were removed to obtain sterile immature embryos. The immature embryos were inoculated on modified H1 medium supplemented with 2.0 mg/L 2,4-dichlorophenoxyacetic acid (2,4-D; Merck, Darmstadt, Germany) and 2.5 mg/L kinetin (KT; Merck), and cultured in the dark at 23 °C ± 1 °C for embryogenic callus induction. After two months of culture, embryogenic callus with compact, dense structure and milky white translucent appearance was obtained (Figure 12B). The embryogenic callus was maintained by subculture, and materials in good condition with vigorous proliferation were selected for subsequent somatic embryo maturation induction experiments.

### 5.2. Somatic Embryo Maturation Culture

The somatic embryo maturation induction medium (H1) was based on LP basal medium, supplemented with maltose (30 g/L; Sangon Biotech, Shanghai, China), L-glutamine (450 mg/L; Merck), casein hydrolysate (500 mg/L; Merck), myo-inositol (500 mg/L; Merck), ABA (8 mg/L; Sangon Biotech), PEG8000 (120 g/L; Sangon Biotech) and 2-(N-morpholino)ethanesulfonic acid monohydrate (MES; 250 mg/L; Sangon Biotech). Prior to autoclaving, the pH was adjusted to 5.8 with 1 mol/L NaOH or HCl, and gellan gum (4 g/L; Sangon Biotech) was added as a gelling agent. The medium was autoclaved at 121 °C for 20 min. Embryogenic callus was inoculated onto somatic embryo maturation induction medium and cultured in the dark at 23 °C ± 1 °C, with periodic observation and recording of somatic embryo maturation progression.

### 5.3. Somatic Embryo Phenotypic Classification

NE exhibited clearly defined boundaries and distinct morphological structures at all developmental stages. They were translucent at early stages, progressively developed into milky-white embryos, and subsequently differentiated into three or more cotyledons with gradual suspensor elongation, ultimately forming intact cotyledonary embryo structures (Figure 1G–L). Conversely, AE were typified by browning during various maturation stages, including browning of the embryonic head and/or suspensor, hypocotyl callusing, or development of fewer than three cotyledons (Figure 1A–F).

### 5.4. Transcriptome Sequencing and Bioinformatics Analysis

NE and AE samples were collected separately, flash-frozen in liquid nitrogen, and stored at −80 °C for subsequent analysis. Transcriptome sequencing (RNA-seq) was performed by LC-Bio Technology Co., Ltd. (Hangzhou, China). Total RNA was extracted using the TRIzol method, with concentration and purity assessed by NanoDrop (NanoDrop Technologies, Wilmington, DE, USA) and integrity evaluated by Bioanalyzer 2100 (Agilent Technologies, Santa Clara, CA, USA) (RIN > 7.0). PolyA+ mRNA was enriched using oligo(dT) magnetic beads and fragmented with a magnesium ion fragmentation kit at 94 °C for 5–7 min. First-strand cDNA was synthesized using SuperScript II reverse transcriptase, followed by second-strand synthesis catalyzed by DNA polymerase I and RNase H with dUTP incorporation. After end repair, A-tailing, and adapter ligation, target fragments were size-selected using magnetic beads. The labeled strand was digested with UDG enzyme, and the library was amplified by PCR (95 °C for 3 min; 98 °C for 15 s, 60 °C for 15 s, 72 °C for 30 s, 8 cycles; 72 °C for 5 min) to obtain strand-specific libraries of approximately 300 bp ± 50 bp. Paired-end sequencing (PE150) was performed on the Illumina NovaSeq 6000 platform.

Raw sequencing data were processed with Cutadapt (v1.9) to remove adapter contamination, low-quality bases, and reads containing ambiguous nucleotides (N), followed by quality control assessment using FastQC (v0.10.1). High-quality clean reads were de novo assembled using Trinity (v2.15). Transcripts were clustered based on sequence similarity into “gene” clusters, with the longest transcript from each cluster selected as the Unigene sequence. Functional annotation of Unigenes was performed by alignment against Nr, GO, Swiss-Prot, KEGG, and eggNOG databases using DIAMOND (v2.0.15, E-value < 1 × 10^−5^). Gene expression levels were quantified as TPM values using Salmon (v1.9.0), and differentially expressed genes were identified using the R package edgeR (v3.40.2) with the criteria of |log_2_(fold change)| > 1 and FDR < 0.05. The data has been uploaded to NCBI, with the numbers SRR39976002, SRR39976037, SRR39966278, SRR39950017, SRR39950014 and SRR39949516.

### 5.5. Quantitative Real-Time PCR Validation

Key genes involved in somatic embryogenesis, as well as differentially expressed genes from plant hormone signal transduction, glutathione metabolism, and riboflavin metabolism pathways, were selected from the transcriptome data for qRT-PCR validation. Gene-specific primers were designed and *18S* was used as the internal reference gene. Total RNA was extracted using the KK Fast Plant Total RNA Kit (ZOMANBIO, Beijing, China). Reverse transcription was performed using the HiScript III 1st Strand cDNA Synthesis Kit (+gDNA wiper) (Vazyme, Beijing, China), and cDNA was quantified using the Equalbit Kit (Vazyme). qRT-PCR was conducted using the AceQ^®^ qPCR SYBR^®^ Green Master Mix (Vazyme) on a PTC-100 thermal cycler (Bio-Rad, Hercules, CA, USA ), with a reaction volume of 20 µL and three technical replicates for each sample. The relative expression levels were calculated using the 2^−^ΔΔCt method to verify the reliability of the transcriptome sequencing results. Primer sequences are listed in Supplementary Table S1.

### 5.6. Exogenous Reduced Glutathione and Riboflavin Treatments for Somatic Embryo Maturation Induction

Embryogenic callus with stable proliferation and uniform morphology was selected for maturation induction. In a laminar flow hood, callus was cut into pieces of approximately 0.04 g each. For each treatment, 15 pieces were evenly distributed into three Petri dishes containing solid induction medium (5 pieces per dish). After 60 days of culture, the callus was transferred to fresh medium of the same composition and cultured for an additional 60 days (120 days total).

The number of somatic embryos (including both NE and AE) in each dish was counted. To standardize the data, embryo counts were converted to embryos per gram of initial callus using the following formula: Embryos per gram = Embryos per dish/5 × 25. Rationale: Each Petri dish contained 5 callus pieces, and each piece weighed approximately 0.04 g. Therefore, 1 g of initial callus corresponds to 25 pieces (1 ÷ 0.04 = 25). Dividing the embryo count per dish by 5 gives the average number of embryos per callus piece; multiplying by 25 converts this value to the number of embryos per gram of initial callus. This calculation was applied to both normal embryos and abnormal embryos. The mean value per dish was treated as an independent replicate, with at least three biological replicates for each treatment. The percentage of normal embryos was calculated as (number of normal embryos/total number of embryos) × 100%.

Exogenous treatments were based on H1 medium, including: reduced glutathione (GSH) at 0 (control), 0.1, 0.5, 1.0, and 5.0 mM; and riboflavin at 0 (control), 2.5, 5.0, 7.5, and 10.0 µM. All cultures were maintained at 23 °C ± 1 °C in continuous darkness. Somatic embryo development was observed and recorded periodically. After 120 days, the number of normal embryos, abnormal embryos, and the proportion of normal embryos were statistically analyzed for each treatment group.

### 5.7. Orthogonal Combination Experiment of GSH and Riboflavin for Somatic Embryo Maturation Induction

Based on the single-factor experimental results, two GSH concentrations (0.1 mM and 0.5 mM) and two riboflavin concentrations (5.0 µM and 7.5 µM) were selected for a 2 × 2 orthogonal combination, establishing four treatment groups. Embryogenic callus was inoculated onto each treatment medium following the method described in Section 5.6. After culture completion, the number of normal embryos, abnormal embryos, and the proportion of normal embryos were statistically analyzed for each treatment group (Table 1).

### 5.8. Data Processing and Statistical Analysis

All experiments were conducted with at least three biological replicates. Statistical analyses were performed using SPSS 26.0 (IBM, Chicago, IL, USA), and graphical presentations were generated using GraphPad Prism 9.0 (GraphPad Software, San Diego, CA, USA). One-way analysis of variance (ANOVA) was employed as the primary statistical method according to the experimental design. The significance level was set at α = 0.05.

## Figures and Tables

**Table 1:** Orthogonal experimental design table.

Group	GSH (mM)	Riboflavin (µM)
1	0.1	5
2	0.1	7.5
3	0.5	5
4	0.5	7.5

## Data Availability

Data is contained within the article.

## Supplementary Materials

The following supporting information can be downloaded at: https://www.mdpi.com/article/10.3390/plants15182768/s1.

## Abbreviations

SE	somatic embryogenesis
NE	normal embryos
AE	abnormal embryos
GSH	glutathione
GSSG	oxidized glutathione
PEG	polyethylene glycol
FMN	flavin mononucleotide
FAD	flavin adenine dinucleotide
ROS	reactive oxygen species
ABA	abscisic acid
JA	jasmonate
GA	gibberellin
CK	cytokinin
BR	brassinosteroid
SA	salicylic acid
NPA	N-1-naphthylphthalamic acid
2,4-D	2,4-dichlorophenoxyacetic acid
BA	6-benzylaminopurine
KT	kinetin
IAA	1H-Indole-3-acetic acid
AC	Activated Charcoal
EC	embryogenic callus
